# SERPINB4 Promotes Keratinocyte Inflammation via p38MAPK Signaling Pathway

**DOI:** 10.1155/2023/3397940

**Published:** 2023-03-21

**Authors:** Yanan Zhang, Luling Wang, Xiaoying Sun, Fulun Li

**Affiliations:** ^1^Shanghai Municipal Hospital of Traditional Chinese Medicine, Shanghai University of Traditional Chinese Medicine, Shanghai 200071, China; ^2^Yueyang Hospital of Integrated Traditional Chinese and Western Medicine, Shanghai University of Traditional Chinese Medicine, Shanghai 200437, China; ^3^Institute of Dermatology, Shanghai Academy of Traditional Chinese Medicine, Shanghai 201203, China

## Abstract

Psoriasis is a chronic inflammatory skin disease characterized by infiltration of inflammatory cells and excessive proliferation of epidermal keratinocytes. SERPINB4, as a serine protease inhibitor, has been clearly expressed in the skin lesions and serum of patients with psoriasis, but the specific mechanism of action is not yet clear. Here, we showed that SERPINB4 expression was increased in skin lesions from the imiquimod (IMQ)-treated mice and M5-(a mixture of five proinflammatory cytokines: IL-17A, IL-22, IL-1*α*, oncostatin M, and TNF-*α*) treated human immortalized keratinocyte (HaCaT). Knockdown of SERPINB4 by short hairpin RNA attenuated the M5-induced keratinocyte inflammation. Conversely, lentiviral expression of SERPINB4 promoted keratinocyte inflammation. Finally, we observed that SERPINB4 stimulation activated the p38MAPK signaling pathway. Taken together, these results suggest that SERPINB4 has a critical role in psoriasis pathogenesis.

## 1. Introduction

Psoriasis, an immune-mediated chronic inflammatory skin disease, is characterized by epidermal hyperproliferation and infiltration of inflammatory cells [1]. As a chronic relapsing and remitting inflammatory skin disease, it affects 2%–3% of the world's population [2]. As the prolonged erythematous scaly patches or plaques in any skin site, it seriously affects the quality of life of the patient [3]. Although the pathogenesis is unclear, hyperactive keratinocytes and immune cells are a key pathological process during psoriasis development, and the crosstalk between these cells contributes to the pathological phenotype.

Squamous cell carcinoma antigen 2 (SCCA2, SERPINB4), as one of the squamous cell carcinoma antigens, has been used as a tumor marker of some squamous cell carcinomas to predict the pathological grade, stage, recurrence, and response to radiotherapy and chemotherapy [4]. SERPINB4 can promote the migration [5] and invasion [6] of cancer cells, and participate in the process of cell senescence [7] and inflammatory reaction [8].

Further, accumulating research has indicated that SERPINB4 is upregulated in many inflammatory skin diseases including psoriasis [9, 10] and atopic dermatitis [11, 12], and serum SERPINB4 level is positively correlated with clinical severity of patients. However, the specific mechanism of action in these diseases is still unknown.

In the data obtained in our previous study, we found that SERPINB4 expression was associated with increased psoriasis severity [13]. In this study, we first discovered that the expression of SERPINB4 was upregulated in skin lesions of IMQ-induced psoriasis-like mice and M5-induced psoriasiform cell model, and then examined the biological function of SERPINB4 by knockdown and overexpression approaches in vitro. Our results showed that SERPINB4 may promote keratinocyte inflammation via activation of p38MAPK in psoriasis. Thus targeting SERPINB4 may represent a potent strategy in psoriasis treatment.

## 2. Materials and Methods

### 2.1. Experimental Animals

The male BALB/c mice, 6–8 weeks, were purchased from Shanghai Slake Laboratory Animal Co., Ltd. (Shanghai, China). Animal experiments were performed at the Animal Research Center of Shanghai Municipal Hospital of Traditional Chinese Medicine, Shanghai University of Traditional Chinese Medicine under the National Institutes of Health regulations. Our protocols were ratified by the medical ethics committee of Shanghai municipal Hospital of Traditional Chinese Medicine, Shanghai University of Traditional Chinese Medicine. The mice were housed at the Animal Research Center of Shanghai Municipal Hospital of Traditional Chinese Medicine, Shanghai University of Traditional Chinese Medicine under typical environmental surroundings at 25 ± 0.5°C and 12 hr light–dark cycle with ad libitum access to standard laboratory water and food.

### 2.2. Animal Experiments

The experimental mice received a daily topical dose of 62.5 mg of commercially available IMQ cream (5%; Sichuan Mingxing Pharmaceutical Co., Ltd., Chengdu, China) on the shaved back and the right ear for 6 consecutive days. The Control mice were treated similarly with a control vehicle cream (Nanchang Baiyun Pharmaceutical Co., Ltd., Nanchang, China). On the days indicated, the ear thickness of the right ear was measured in duplicate using digital calipers (Mitutoyo). On the seventh day, all mice were killed and skin specimens were collected and inspected.

### 2.3. Cell Culture

The HaCaT cells were cultured in DMEM containing 10% heat-inactivated fetal bovine serum, penicillin (100 U/ml), and streptomycin (100 mg/ml) at 37°C in a humidified atmosphere with 5% CO_2_.

### 2.4. Induction of the In Vitro Psoriatic Model

HaCaT cells were stimulated with 10 ng/ml recombinant IL-17A (Prospec Protein Specialists, CYT-250), OSM (Prospec Protein Specialists, CYT-231), TNF-*α* (Prospec Protein Specialists, CYT-223), IL-22 (Prospec Protein Specialists, CYT-328), and IL-1*α* (Prospec Protein Specialists, CYT-253) in combination (named M5 combination) in DMEM supplemented with 2% (v:v) FBS, to recapitulate numerous features of psoriasis.

### 2.5. Plasmids and Reagents

Flag-human SERPINB4 was cloned into the pcDNA3.1 plasmid. Short hairpin RNA of (SERPINB4) and short hairpin RNA of GFP (shControl) constructs described as previously were cloned into the pLKO.1 vector (Addgene). shRNA targeting sequences used are sh-GFP: 5′-tacaacagccacaacgtctat-3′; shS-ERPINB4#1 : 5-gcagaagcttgaagagaaact-3′; sh-SERPIN4#2 : 5′-ggacaagtttgcagaatatga-3′.

### 2.6. Short Hairpin RNA or Lentiviral Expression

Constructs were transfected along with pMD2.G and psPAX2 into 293T cells. Forty-eight and seventy-two hours after initial transfection, viral supernatant was collected, filtered, supplemented with polybrene (10 *µ*g/ml), and used to infect HaCaT cells. Forty-eight hours after the last infection, cells were selected with appropriate antibiotics. Transduced cells were selected with puromycin or G418.

### 2.7. Enzyme-Linked Immunosorbent Assay (ELISA)

To detect the levels of CXCL8 in the cell culture supernatant, the cell culture supernatants were collected, and the CXCL8 level was measured using CXCU8 ELISA kit (Thermo Fisher Scientific, 88-8086), according to the manufacturer's instructions.

### 2.8. Western Blot

The total protein of mice skin or the cells was prepared using RIPA lysis buffer (Beyotime, Beijing, China) following the manufacturer's protocols. Equal amounts of protein were then loaded on SDS–PAGE gels, and after electrophoretic separation, they were transferred electrophoretically to a PVDF membrane (Millipore, USA). After blocking with 5% milk in TBST, the membrane was incubated overnight with primary antibody (1 : 1,000) at 4°C. After washing and incubation, the membrane was incubated with secondary antibody (1 : 2,000) in TBST. Protein expression levels were detected by enhanced chemiluminescence (ECL) Plus reagent (Millipore, Billerica, MA, USA) in Image Pro Plus software (Media Cybernetics, Rockville, MD). The following primary antibodies were used in this study: anti-SERPINB4 (Abcam, ab197096, 1 : 1,000 dilution), anti-*β*-tubulin (CST, 2146, 1 : 1,000 dilution), anti-phospho-p38MARK (CST, 4511, 1 : 1,000 dilution), and anti-p38MAPK (CST, 8690, 1 : 1,000 dilution).

### 2.9. Real-Time PCR

The total RNA of cells was isolated using Trizol (Takara, Kyoto, Japan) and subsequent chloroform–isopropanol–ethanol purification, and cDNA was synthesized with a PrimeScript RT Reagent Kit (Takara) according to the manufacturer's protocols and used as the template for quantitative real-time PCR. Quantitative real-time PCR was performed using SYBR premix EX TaqI (Takara, Japan) on a Chromo4 continuous fluorescence detector with a PTC-200 DNA Engine Cycler (Bio-Rad, Hercules, CA, USA). Relative quantification was performed according to the 2^−*ΔΔ*Ct^ method, with normalization for ACTB expression. Primer sequences were as follows: human SERPINB4 (forward: 5′-gtcgatttacacttacctcgg-3′, reverse: 5′-gccttgtgtaggactttagatact-3′); humanCXCL1 (forward: 5′-cccaaaccgaagtcatagcca-3′, reverse: 5′-ttggatttgtcactgttcagcatc-3′); humanCXCL2 (forward: 5′-cccaaaccgaagtcatagcca-3′, reverse:5′-gttggatttgccatttttcagc-3′); humanCCL20 (forward: 5′-tgctgtaccaagagtttgctc-3′, reverse: 5′-cgcacacagacaactttttcttt-3′); humanS100A7 (forward: 5′-acgtgatgacaagattgagaagc-3′, reverse: 5′-gcgaggtaatttgtgcccttt-3′); humanACTB (forward: 5′-catgtacgttgctatccaggc-3′, reverse: 5′-ctccttaatgtcacgcacgat-3′); mouse serpinb3a (forward:5′-ttggctgaacaagaacacaag-3′, reverse: 5′-tggcaacaggacaatcatactta-3′); mouse cxcl1 (forward: 5′-actgcacccaaaccgaagtc-3′, reverse: 5′-tggggacaccttttagcatctt-3′); mousecxcl2 (forward: 5′-ccaaccaccaggctacagg-3′, reverse: 5′-gcgtcacactcaagctctg-3′); mouse ccl20 (forward: 5′-actgttgcctctcgtacataca-3′, reverse: 5′-gaggaggttcacagccctttt-3′); mouse s100a7a (forward: 5′-tgctcttggatagtgtgcctc-3′,reverse: 5′-gctctgtgatgtagtatggctg-3′); mouse actb (5′-gtgacgttgacatccgtaaaga-3′, reverse: 5′-gccggactcatcgtactcc-3′). Results were from three independent experiments.

### 2.10. Statistical Analysis

All statistical analyses were performed with the GraphPad Prism 8.0 software (GraphPad Software). Data from at least three independent experiments are presented as the mean ± standard deviation (SD). Student *t*-test (comparisons between two groups) or one-way ANOVA followed by Kruskal–Wallis test (more than two groups) were used to analyze the statistical significance. *p* < 0.05 was considered to be statistically significant.

## 3. Results

### 3.1. Serpinb3a (Mouse Homolog of SERPINB4) Was Induced and Activated in IMQ-Induced Psoriasiform Lesions in Mice

**Figure 1:**
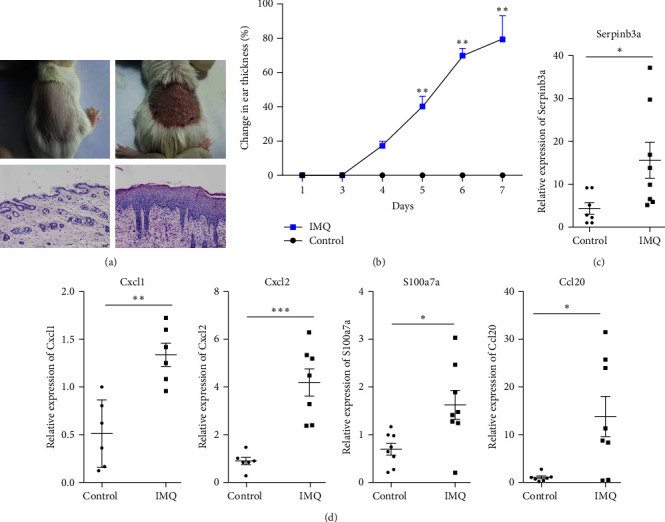
Serpinb3a is highly expressed in imiquimod (IMQ)-induced psoriasis-like mice: (a) phenotypical presentation and H&E staining of IMQ-induced psoriasis-like mice, (b) ear thickness of the right ear was measured on the days indicated, (c) Serpinb3a expression in IMQ-treated back skins detected by real-time PCR, (d) mRNA expression of Cxcl1, Cxcl2, S100a7a, Ccl20 in back skins examined by real-time PCR. The results are represented as the mean ± SD, *n* = 7.  ^*∗*^*p* < 0.05,  ^*∗∗*^*p* < 0.01, and  ^*∗∗∗*^*p* < 0.001 vs. control, which indicates a statistically significant difference.

Given that increased SERPINB4 expression was associated with psoriasis severity, we are committed to discussing its role in the pathogenesis of psoriasis. First, we established a psoriasis-like skin disorder in BALB/c mice using IMQ treatment, according to previous reports [14]. We applied IMQ cream on the shaved back skin and right ear of BALB/c mice for 6 consecutive days. As shown in Figure 1(a), mice treated daily with control cream did not show any sign of inflammation, but in IMQ-treated mice, hematoxylin–eosin staining of sections of the back skin showed that the thickness of the epidermis increased and inflammatory cells infiltrated obviously. Daily IMQ treatment of the right ear of the mice led to a significant increase in ear thickness, the ear changes were statistically significant on the 5, 6, and 7 days (the fifth day: IMQ: mean ± SD: 40.13 ± 13.36, Control: mean ± SD: 0 ± 0, *p* < 0.01; the sixth day: IMQ: mean ± SD: 69.68 ± 9.499, Control: mean ± SD: 0 ± 0, *p* < 0.01; the seventh day: IMQ: mean ± SD: 79.24 ± 30.72, Control: mean ± SD: 0 ± 0, *p* < 0.01) (Figure 1(b)). Using real-time PCR, we found that serpinb3a expression in the back skin of IMQ-treated mice (mean ± SD: 15.62 ± 11.89) was significantly upregulated compared with that of control mice (mean ± SD: 4.367 ± 3.559, *p* < 0.05) (Figure 1(c)). The IMQ-treated mice showed raised expression of T17 cell chemotactic factor (Ccl20), neutrophil chemotactic factors (Cxcl1, Cxcl2), and antimicrobial peptide S100a7a in skin lesions compared with the control mice (Ccl20: IMQ: mean ± SD:13.89 ± 11.86, Control: mean ± SD: 1.019 ± 0.840, *p* < 0.05; Cxcl1: IMQ: mean ± SD: 1.336 ± 0.297, Control: mean ± SD 0.511 ± 0.354, *p* < 0.01; Cxcl2: IMQ: mean ± SD: 4.194 ± 1.539, Control: mean ± SD: 0.882 ± 0.379, *p* < 0.001; S100a7a: IMQ: mean ± SD: 1.629 ± 0.855, Control: mean ± SD: 0.698 ± 0.344, *p* < 0.05) (Figure 1(d)). Taken together, these results suggested that serpinb3a may be related to the pathogenesis of psoriasis.

### 3.2. SERPINB4 Was Upregulated in Cell Model of Psoriasis

**Figure 2:**
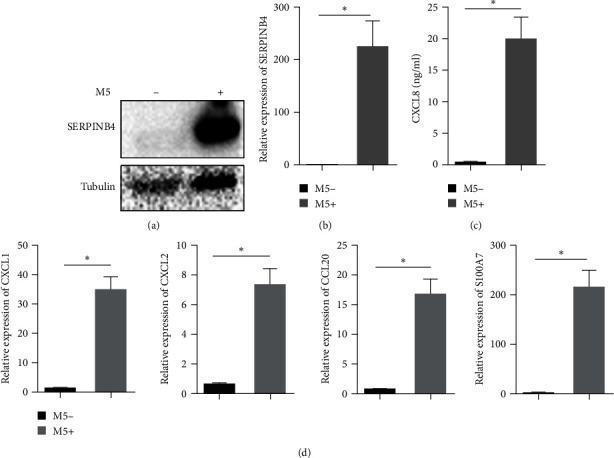
SERPINB4 was upregulated in cell model of psoriasis: (a) SERPINB4 protein expression detected by western blotting stimulated with or without 10 ng/ml M5 for 48 hr, (b) SERPINB4 mRNA expression detected by real-time PCR stimulated with or without 10 ng/ml M5 for 24 hr, (c) CXCL8 in the culture medium by ELISA kit at 48 hr, (d) mRNA levels of the indicated genes were assessed by real-time PCR at 24 hr. All results are represented as the mean ± SD.  ^*∗*^*p* < 0.05 vs. control (M5−), which indicates a statistically significant difference.

Tumor necrosis factor-alpha (TNF-*α*), interleukin-1 alpha (IL-1*α*), interleukin-17A (IL-17A), interleukin-22 (IL-22), and oncostatin M (OSM) are closely associated with the pathogenesis of psoriasis [15–18]. The combination of TNF-*α*, IL-1*α*, IL-17A, IL-22, and OSM (termed as M5) has been shown to induce psoriasis-like changes, including cell inflammation and increased cell proliferation in cultured keratinocytes [19]. In this study, the synergistic action of M5 indeed raised the mRNA of chemokines (CXCL1, CXCL2, and CCL20), antimicrobial peptide (S100A7) and the secretion of CXCL8 (CXCL1: M5+: mean ± SD: 34.920 ± 8.530, M5−: mean ± SD: 1.348 ± 0.250, *p* < 0.05; CXCL2: M5+: mean ± SD: 7.329 ± 2.232; M5−: mean ± SD: 0.581 ± 0.292, *p* < 0.05; CCL20: M5+: mean ± SD:16.670 ± 5.246; M5−: mean ± SD: 0.673 ± 0.259, *p* < 0.05; S100A7: M5+: mean ± SD: 215.8 ± 69.75; M5−: mean ± SD: 2.393 ± 1.361, *p* < 0.05; CXCL8: M5+: mean ± SD: 19.770 ± 3.618; M5−: mean ± SD: 0.330 ± 0.214, *p* < 0.05) (Figures 2(c) and 2(d)) Meanwhile, M5 significantly induced SERPINB4 expression in HaCaT cell at both mRNA and protein levels (SERPINB4: M5+: mean ± SD: 224.9 ± 96.77; M5−: mean ± SD: 0.6109 ± 0.5270, *p* < 0.05) (Figures 2(a) and 2(b)). All these results showed that upregulation of SERPINB4 expression may be related to keratinocyte inflammation.

### 3.3. SERPINB4 Knockdown Inhibited Keratinocyte Inflammation

**Figure 3:**
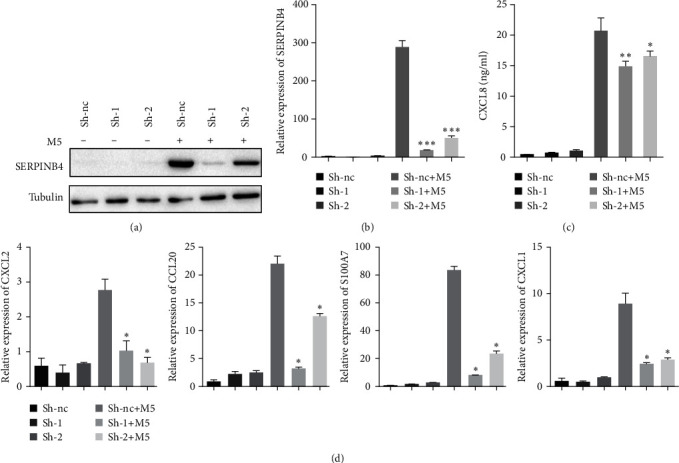
SERPINB4 knockdown inhibited keratinocyte inflammation: (a) SERPINB4 knockdown levels in human HaCaT after transfection with shRNA (Sh-1 or Sh-2) or vector (Sh-nc) by western blotting with or without 10 ng/ml M5 for 48 hr, (b) SERPINB4 knockdown levels in human HaCaT cells after transfection with shRNA or vector by real-time PCR with or without 10 ng/ml M5 for 24 hr, (c) CXCL8 in the culture medium by ELISA kit at 48 hr, (d) mRNA levels of the indicated genes were assessed by real-time PCR at 24 hr. The results are represented as the mean ± SD.  ^*∗*^*p* < 0.05,  ^*∗∗*^*p* < 0.01 and  ^*∗∗∗*^*p* < 0.001 vs. control (Sh-nc), which indicates a statistically significant difference.

To determine the effect of SERPINB4 on keratinocyte inflammation, we stably transduced HaCaT cells with lentivirus harboring short hairpin RNA against SERPINB4 (Sh-1, Sh-2) and a scramble shRNA sequence (Sh-nc). As shown in Figures 3(a) and 3(b), at basic level, SERPINB4 mRNA and protein expressions were barely detectable in HaCaT cell, but SERPINB4 expression was significantly upregulated after M5 stimulation. The level of Sh-SERPINB4 (Sh-1 or Sh-2) protein and mRNA was significantly lower than that of Sh-nc. (SERPINB4: Sh-nc+M5 : 288.9 ± 17.02 vs. Sh-1+M5 : 18.32 ± 0.980 vs. Sh-2+M5 : 50.7 ± 5.022, *p* < 0.05). Next, we found that knockdown SERPINB4 expression inhibited the mRNA of chemokines (CXCL1, CXCL2, and CCL20) (CXCL1: Sh-nc+M5 : 8.981 ± 1.071 vs. Sh-1+M5 : 2.383 ± 0.2168 vs. Sh-2+M5 : 2.891 ± 0.175, *p* < 0.05; CXCL2: Sh-nc+M5 : 2.776 ± 0.628 vs. Sh-1+M5 : 1.022 ± 0.560 vs. Sh-2+M5 : 0.677 ± 0.341, *p* < 0.05; CCL20: Sh-nc+M5 : 22.11 ± 2.745 vs. Sh-1+M5 : 3.195 ± 0.475 vs. Sh-2+M5 : 12.56 ± 1.040, *p* < 0.05) (Figure 3(d)), antimicrobial peptides (S100A7) (S100A7: Sh-nc+M5 : 83.90 ± 5.039 vs. Sh-1+M5 : 8.472 ± 0.069 vs. Sh-2+M5 : 23.36 ± 4.760, *p* < 0.05) (Figure 3(d)) and the secretion of CXCL8 (Sh-nc+M5 : 20.79 ± 3.648 vs. Sh-1+M5 : 14.88 ± 1.784 vs. Sh-2+M5 : 16.47 ± 1.926, *p* < 0.05) in M5-induced HaCaT (Figure 3(c)).

### 3.4. SERPINB4 Overexpression Promoted Keratinocyte Inflammation

**Figure 4:**
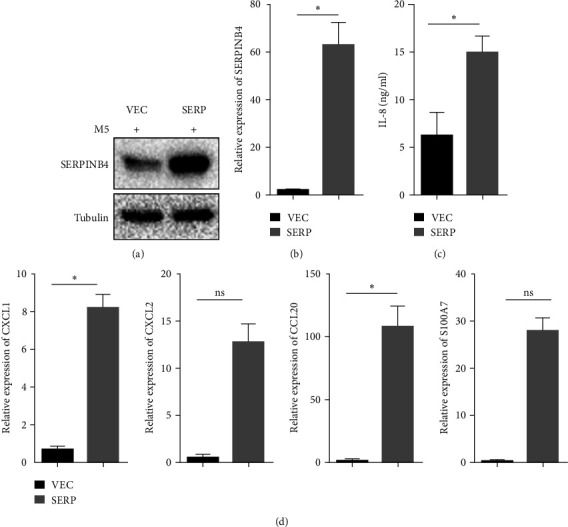
SERPINB4 overexpression promoted keratinocyte inflammation: (a) SERPINB4 overexpression levels in HaCaT cells after transfection with overexpressing lentivirus (SER) or control (VEC) by western blotting with 2.5 ng/ml M5 for 48 hr, (b) SERPINB4 overexpression level in HaCaT cells after transfection with overexpressing lentivirus (SERP) or control (VEC) by real-time PCR, (c) CXCL8 in the culture medium by ELISA kit at 48 hr, (d) mRNA levels of the indicated genes were assessed by real-time PCR at 24 hr. The results are represented as the mean ± SD.  ^*∗*^*p* < 0.05 vs. control (VEC), which indicates a statistically significant difference, ns indicates no significant.

Next, we constructed SERPINB4 lentiviral overexpression cell line (Figures 4(a) and 4(b)). Overexpression of SERPINB4 (SERP) promoted the mRNA of chemokines (CXCL1, CXCL2, and CCL20) (CXCL1: VEC: 0.728 ± 0.281, SERP: 8.225 ± 1.176, *p* < 0.05; CCL20: VEC: 1.711 ± 2.132, SERP: 108.4 ± 32.51, *p* < 0.05), antimicrobial peptides (S100A7) and the secretion of CXCL8 (VEC: CXCL8 : 6.162 ± 2.470, SERP: 14.88 ± 1.784, *p* < 0.05) compared with that of control (VEC) in M5-treated HaCaT cell (Figures 4(c) and 4(d)).

### 3.5. SERPINB4 Activated p38 MAPK Pathway

**Figure 5:**
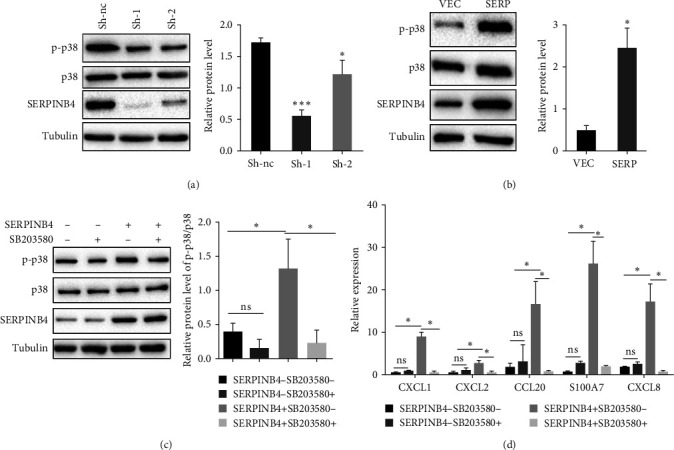
SERPINB4 activated p38MAPK pathway in HaCaT: (a) levels of phospho-p38, p38 in HaCaT after transfection with SERPINB4 (Sh-1 or Sh-2) or vector (Sh-nc) with 10 ng/ml M5 for 12 hr analyzed by Western blot analysis, (b) levels of phospho-p38, p38 in HaCaT after transfection with overexpressing lentivirus (SER) or Control (VEC) with 2.5 ng/ml M5 for 12 hr analyzed by Western blot analysis, (c) levels of phospho-p38, p38 in HaCaT after transfection with overexpressing lentivirus (SER) or Control (VEC) with 2.5 ng/ml M5 for 12 hr before exposure to 10 *μ*M SB203580 (p38 inhibitor) analyzed by Western blot analysis, (d) mRNA expression of CXCL1, CXCL2, S100A7, CCL20, and CXCL8 examined by real-time PCR. The results are represented as the mean ± SD.  ^*∗*^*p* < 0.05 and  ^*∗∗∗*^*p* < 0.001 (Shnc or VEC), which indicates a statistically significant difference.

To elucidate the molecular mechanism of the effect of SERPINB4 on keratinocyte inflammation, we first analyzed the effect of SERPINB4 on the p38 MAPK signaling pathway. Because previous studies have shown that M5 activated p38MAPK signaling pathway in psoriasis and activation of p38MAPK pathway contributed to the regulation of the production of inflammation mediators [20–22]. As shown in Figure 5(a), knockdown of SERPINB4 decreased the phosphorylation of p38 compared with the control cell in M5-induced HaCaT cell (Sh-nc+M5 : 1.718 ± 0.078 vs. Sh-1+M5 : 0.552 ± 0.099 vs. Sh-2+M5 : 1.214 ± 0.226, *p* < 0.05). On the contrary, overexpression of SERPINB4 significantly increased the phosphorylation of p38 in M5-treated HaCaT cell (VEC: 0.493 ± 0.108, SERP: 2.447 ± 0.481, *p* < 0.05) (Figure 5(b)). Next, we pretreated HaCaT cell with SB203580, an inhibitor of p38MAPK, before transfected with SERPINB4. As shown in Figure 5(c), the phosphorylation level of p38 was significantly diminished by SB203580 (SERPINB4+SB203580−:1.312 ± 0.437, SERPINB4+SB203580+: 0.226 ± 0.188). Meanwhile, the mRNA expression of CXCL1, CXCL2, CCL20, S100A7, and CXCL8 was inhibited when incubated with SB203580 (CXCL1: SERPINB4+SB203580−: 8.981 ± 1.07, SERPINB4+SB203580+: 0.566 ± 0.290; CXCL2: SERPINB4+SB203580−: 2.776 ± 0.627, SERPINB4+SB203580+: 0.581 ± 0.291; CCL20: SERPINB4+SB203580−: 16.668 ± 5.246, SERPINB4+SB203580+: 0.771 ± 0.297; S100A7: SERPINB4+SB203580−: 26.153 ± 5.326, SERPINB4+SB203580+:1.850 ± 0.220; CXCL8: SERPINB4+SB203580−: 17.168 ± 4.292, SERPINB4+SB203580+: 0.695 ± 0.313) (Figure 5(d)). Taken together, these in vitro and in vivo results suggested SERPINB4 promotes keratinocyte inflammation via the p38MAPK signaling pathway.

## 4. Discussion

Squamous cell carcinoma antigens (SCCAs) are members of the serpin family of endogenous protease inhibitors [23]. SCCA is encoded by two highly homologous genes (SCCA1 and SCCA2), which are arranged in tandem at chromosome 18q21.3 [24]. SCCA1 and SCCA2 belong to class B serine family protease inhibitors (SERPINs), which were later named SERPINB3 and SERPINB4 [25]. Tissue distribution studies have shown that SERPINB3 and SERPINB4 are coexpressed in the suprabasal layers of the stratified squamous epithelium of the tongue, esophagus, uterine cervix, vagina, tonsil, Hassall's corpuscles of thethymus, and some areas of the skin [26].

SERPINB3/SERPINB4 has been found to be upregulated in a variety of cancer tissues, including esophageal cancer [27], cervical cancer [28], head and neck cancer [29], liver cancer [30], breast cancer [31], and so on. Studies have found that SERPINB3/SERPINB4 can promote the migration and invasion ability of cancer cells, and participate in the process of cell aging and inflammatory response.

In recent years, increasing evidence has implicated that serine proteases are critical for epidermal barrier homeostasis and their aberrant expression or activity is associated with chronic skin diseases. Elevated levels of the serine protease inhibitors SERPINB3 and SERPINB4 are seen in patients with atopic dermatitis and psoriasis, further clinical studies found that SERPINB4 is closely associated with the clinical severity of these two diseases. However, their mechanistic role in these skin diseases is unknown. In this study, we found that SERPINB4 expression was upregulated in skin from IMQ-induced psoriasis-like mice as well as in M5-induced psoriasiform cell model. Silencing SERPINB4 expression using shRNA-expressing lentivirus vector in vitro reduced inflammation in M5-induced psoriasiform cell model. Next, we further proved that p38 MAPK is an important signaling pathway mediating the effects of SERPINB4 on keratinocyte inflammation. Taken together, these in vitro and in vivo results pointed toward a potential role of SERPINB4 in the pathogenesis of psoriasis.

Guilloteau et al. [19] found stimulation keratinocytes with a combination of five cytokines (M5) induced inflammation that recapitulates some features of psoriasis including increased cell inflammation and cell proliferation in vitro and in vivo. In this study, we also found that treatment with M5 induced mRNA levels of chemokines (CXCL1, CXCL2, and CCL20) and antimicrobial peptide (S100A7) and the secretion of CXCL8 in HaCaT cell. Meanwhile, we further found that M5 treatment can led to upregulation of SERPINB4 mRNA and protein expression. To further confirm whether some connection between SERPINB4 and inflammation or not in psoriatic keratinocytes, we constructed SERPINB4 silence and overexpression cell lines. We found that knockdown of SERPINB4 inhibited cell inflammation. Conversely, overexpression of SERPINB4 promoted keratinocytes inflammation. These results indicated that SERPINB4 promoted keratinocyte inflammation in M5-treated HaCaT cell.

In the process of Ras-associated cytokine production and tumorigenesis, Catanzaro et al. [32] showed that endogenous SERPINB4 can promote the secretion of cytokine IL-6. Overexpression of SERPINB4 in human keratinocytes promoted the secretion of IL-1*α* and IL-6 cytokines [33]. In this study, we demonstrated that SERPINB4 stimulated expression of chemokines CXCL1, CXCL2, CXCL8, and antimicrobial peptides (S100A7) in M5-induced HaCaT cell.

How SERPINB4 regulates inflammation in M5-induced psoriasis-like keratinocyte is still unknown. Previous studies have reported that MAPK was an important inflammation signal pathway in psoriasis. Excessive activation of the MAPK (p38, JNK, and ERK1/2) signaling pathway has a key role in regulating the production of inflammatory mediators in psoriasis [20–23]. And p38 MAPK signaling pathway was activated in M5-induced karatinocytes [34, 35]. We speculated that SERPINB4 may promote inflammation through p38MAPK pathway in the process of psoriasis-like inflammation. Indeed, silencing SERPINB4 reduced the phosphorylation level of p38MAPK in keratinocyte. In summary, SERPINB4 expression was upregulated in patients with psoriasis as well as in vivo and in vitro models of psoriasis. SERPINB4 promoted keratinocyte inflammation, which was associated with activation of p38MAPK signaling pathway.

## 5. Conclusion

**Figure 6:**
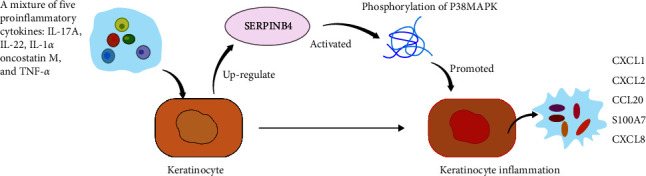
M5 (a mixture of five cytokines: IL-17A, IL-22, IL-1*α*, oncostatin M, and TNF-*α*) stimulated HaCaT cell recapitulating some features of psoriasis including increased cell inflammation and cell proliferation, in this process, SEEPINB4 was activated, which promoted the phosphorylation of p38, to further promote the release of chemokines CXCL1, CXCL2, CCL20, CXCL8, and antimicrobial peptide S100A7, and so on.

This study suggested that SERPINB4 promoted keratinocyte inflammation through p38MAPK signal pathway (Figure 6). SERPINB4 expression was upregulated in skin lesions of IMQ-induced psoriasis-like mice and M5 induced psoriasiform cell model. We confirmed SRPINB4 promoted cell inflammation through SERPINB4 knockdown and overexpression mehtods. Furthermore, SERPINB4 promoted cell inflammation via the p38MAPK pathway. Taken together, our results suggested that SERPINB4 may be implicated in the pathogenesis of psoriasis.

## Data Availability

The data used to support the findings of this study are included within the article.
